# Study on Bingham fractional damage model of backfill material under different moisture content conditions

**DOI:** 10.1371/journal.pone.0295254

**Published:** 2024-01-19

**Authors:** Junguang Wang, Song Yang, Yanming Qi, Yiran Cong

**Affiliations:** 1 School of Mechanics and Engineering, Liaoning Technical University, Fuxin, P R China; 2 Liaoning Province Nonferrous Geology 101 Team Co. Ltd, Fushun, P R China; 3 Qingdao Feiyang Human Resources Development Co. Ltd, Qingdao, P R China; Jamia Millia Islamia, INDIA

## Abstract

Filling mining technology is an important representative technology to realize green and low-carbon mining. The backfill materials have distinct rheological characteristics under the long-term action of formation loads and groundwater seepage. In order to study the creep characteristics of backfill materials under different moisture contents and reveal their aging-mechanical properties, based on the Riemann-Liouville fractional calculus and damage mechanics theory, the fractional element and damage variables are introduced to improve the traditional Bingham model, and the fractional Bingham creep damage model is proposed. Based on the experimental data of gangue cemented backfill under different moisture content, the parameters of the creep model are obtained by using user-defined function fitting and the least square method. The results show that the improved Bingham fractional creep damage model can describe the whole creep process of backfill materials under different moisture contents, and the rationality of the model is verified. Compared with the traditional Bingham model, the fitting degree of the Bingham fractional creep damage model is higher, which solves the problem that the traditional Bingham model cannot describe the nonlinear creep stage. Model parameter *α* and *ξ* increase with the increase of axial stress and moisture content. Under the same moisture content, *η* gradually increases with the increase of axial stress. This work has a certain reference significance for studying the mechanical properties and creep constitutive model of backfill materials containing water.

## Introduction

**Fig 1:**
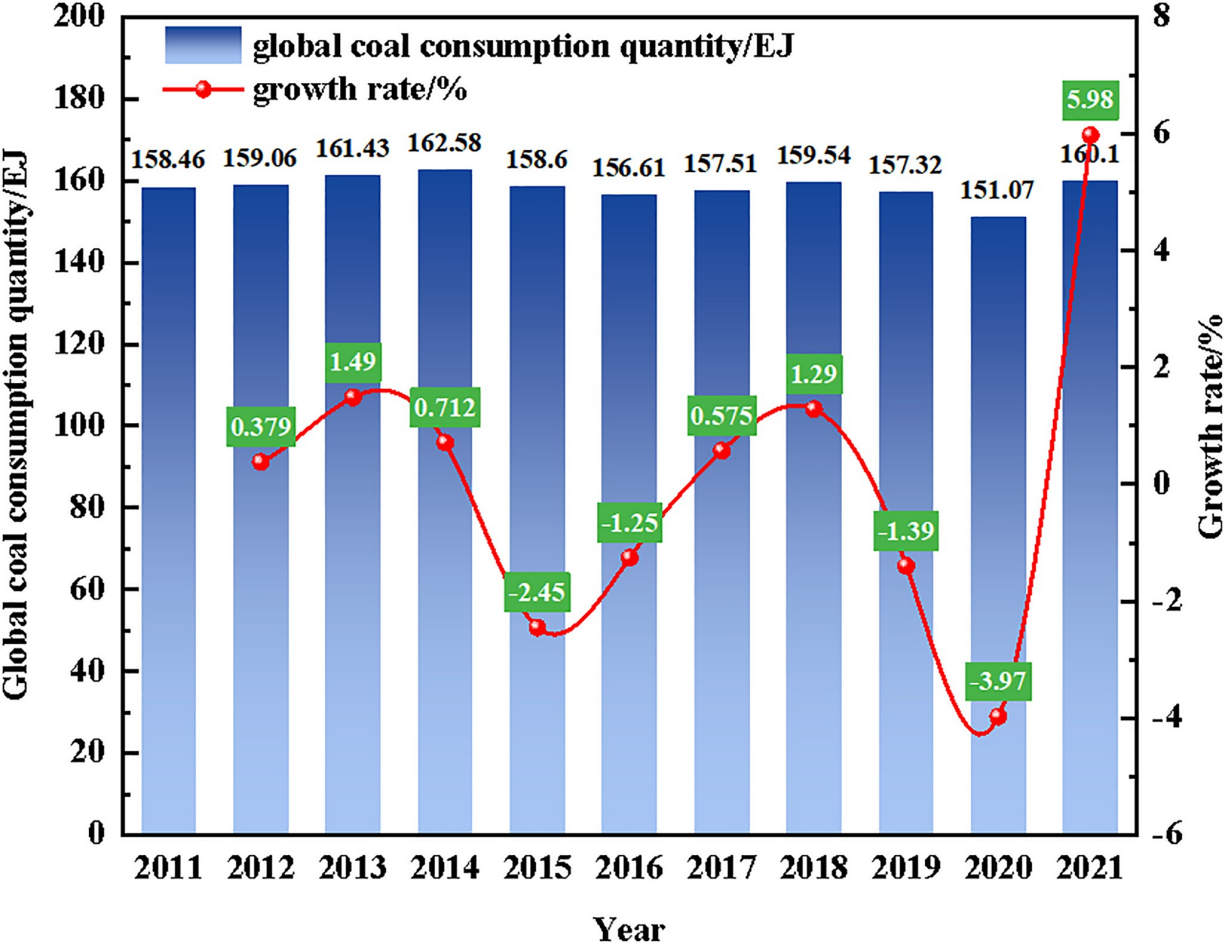
Global coal consumption quantity [1].

Coal is an important basic energy for Chinese industrial economy and social development. It can be seen from Fig 1 that annual global coal consumption quantity is above 150EJ from 2011 to 2022. And global coal consumption increased by 9.95% in the year 2020 to 2021 [1].

On March 17, 2022, the National Energy Board issued the “2022 energy work guidance” which pointed out that it is necessary to consolidate the foundation of energy supply security, accelerate the green and low-carbon transformation of energy, and enhance the flexibility and resilience of the energy supply chain. Therefore, coal green and low-carbon development is imperative. Coal-based solid waste-filling mining technology is an important representative technology to realize green and low-carbon mining. It has remarkable technical advantages in surface subsidence control, ecological environment protection, mine solid waste disposal and utilization, and green and low-carbon emission reduction [2].

Filling mining technology has been widely used in mining engineering. The roof is supported by backfill materials, which greatly reduces the roof deformation and the ground settlement, and has a good control effect [3]. However, the backfill materials has obvious creep characteristics under the long-term effect of stratum load and groundwater seepage. Therefore, it is of great engineering significance to study the creep characteristics of backfill materials under different moisture contents and reveal its time effect-mechanical properties for the evaluation of long-term stability of backfill materials.

At present, many scholars have done a lot of researches on the mechanical properties of the different backfill materials, and have achieved plenteous research achievements. Through the seepage test of coal gangue materials, Li [4] found that that adding a certain concentration of coal fly ash can achieve a better water barrier. Nie [5] revealed that significant differences in the mechanical properties, acoustic emission (AE) responses, maximum principal strain fields, and ultimate failure modes of CGBS under different static-dynamic combined loading paths. Brett [6] establishes the link among induced curing pressure, multiphysics processes, and interface behavior for FRCC materials through analysis of the state-of-the-art research findings on the FM-ITZ of FRCC materials. Gao [7] utilized industrial graphene oxide (GO) and fly ash (FA) to create a high-performance, low-cost, and environment-friendly material for backfill. Sadat [8] discussed the possibility of utilizing the construction and demolition waste (CDW) as cemented recycled aggregate backfill (CRAB) materials in the underground mining industry. Yang [9] revealed the influence of curing age on the dynamic mechanical properties and deformation and failure characteristics of backfill materials under intermediate strain rate. Ran [10] used acoustic emission technology to monitor the damage evolution of strip and column cemented gangue backfill bodies. Rafat [11, 12] found that the tremendous anti-blast performance of the tubular column filled with concrete commonly known as the CFST column as a wise substitute for the conventional RC column. Zhu [13] studied the influence of different mixing ratios on the rheological properties of cemented gangue backfill, and determined the appropriate dosage range.

However, the above research did not consider the effect of moisture content on the creep characteristics of backfill materials. At present, the research on the effect of moisture content on the creep properties of materials is mostly focused on rock materials. Li [14] deeply analyzed the relationship between coal (rock) permeability and the moisture content through seepage experiments of coal (rock) with different moisture contents. Duan [15] used a heat–fluid–solid triaxial servo seepage device to measure the permeability of coal (rock) under different moisture contents and revealed the effects of the slippage effect, permeability, and moisture content. Chen [16] analyzed the acoustic emission damage characteristics of coal (rock) under hydraulic action through uniaxial compression tests under different moisture contents. Wang, [17, 18] established a creep damage model of water-bearing oil shale. Triaxial creep tests of oil shale at different moisture contents were conducted. The rationality of the model was then verified. According to the variation relationship of sandstone strength, the relative strength criterion of sandstone under hydro-mechanical coupling was established [19].

The backfill materials material is different from the rock material, and their creep characteristics are obviously different, and the creep of backfill materials often shows a strong time effect. At present, there have been few studies on the creep constitutive models of backfill materials containing water. It is of great significance to establish a creep model that can describe the whole creep process of backfill materials containing water. Various creep models, including the Maxwell model [20], Kelvin model [21], Bingham model [22], Burgers model [23] and Nishihara model [24], are currently widely used. The Bingham model includes viscous, elastic, and plastic elements, The Bingham model can well describe the steady creep stage of the backfill materials. Compared with these four classical models, the Bingham model has the advantage of fewer parameters and can describe the instantaneous deformation and steady creep well. However, owing to the limitations of its constitutive equation, it can only describe the linear creep stage and cannot accurately describe the nonlinear creep stage.

A large number of creep phenomena show that the strain state of a point in the material is not only related to the same instantaneous stress state of the point, but also to the whole stress history of the point before this time. By coincidence, the fractional time derivative is actually a differential-integral convolution operator, and the integral term in its definition fully reflects the historical dependence of the development of the system function and is a powerful mathematical tool for modeling processes with strong memory [25]. In recent years, a fractional element based on Fractional Order Theory has been gradually applied to the study of creep models.

Zhang [26] proposed a triaxial creep model of deep coal based on fractional derivative considering the temperature effect for triaxial stress state conditions. Based on the modified Nishihara model, Cheng [27] proposed a nonlinear creep model, considering the viscoelastic-plastic and damage evolution characteristics of the rock. On the basis of the Caputo variable-order fractional derivative, Liu [28] proposed the Caputo variable-order fractional creep model. In order to determine the nonlinear creep characteristics of rock under cyclic loading and unloading conditions, Zhang [29] proposed the nonlinear Kelvin model and damage viscoplastic model. Based on the fractional order theory, Huang [30] established a viscoelastic-plastic model considering the initial damage of coal samples. Gao [31] established a new variable fractional rheological model to describe the full-stage creep behavior of rock.

Based on the above problems, the fractional element [32] proposed previously was introduced in this paper. Its constitutive model has fewer parameters than other models and allows for convenient calculations. The strain of the fractional element has a power function relationship with time, which is consistent with the initial creep stage in the creep process of backfill materials. The model reflects the nonlinear characteristics of the creep phenomenon. To characterize the accelerated creep stage, a viscous element with damage variables was introduced. The above elements were combined with the traditional Bingham model to form an improved Bingham fractional creep model. One- and three-dimensional constitutive equations of the model under three different conditions ($\dot{\varepsilon }(t\to \infty )=0,\sigma <\sigma_{s}$, $\dot{\varepsilon }(t\to \infty )>0,\sigma <\sigma_{s}$, $\dot{\varepsilon }(t\to \infty )>0,\sigma \geq \sigma_{s}$) were deduced, and a simple parameter identification method was created. Based on the experimental data of gangue cemented backfill under different moisture content [33], the parameters of the triaxial creep data under different moisture contents were then identified, and the test curve was compared with the theoretical curve to verify the rationality and effectiveness of the model. Correlations between the parameters of the model and the moisture content were analyzed. The research results can provide an important reference for the study of the influence of moisture content on the mechanical properties of backfill materials and the creep constitutive model.

## Fractional calculus theory

### Fractional calculus

Fractional calculus has evolved on the basis of integer order calculus, evolving many definitions such as Riemann-Liouville, Captuo and Grunwald-Letnikov. Riemann–Liouville fractional calculus [34] is the earliest defined and most complete fractional calculus. The most widely used of these is the Riemann-Liouville fractional calculus. Moreover, the definition of Riemann-Liouville fractional calculus is more rigorous in mathematical expression. To facilitate the model derivation and numerical calculations, Riemann–Liouville fractional calculus was used in this study. The integral of order *α* of a function *f*(*x*) is defined as follows:

$$\frac{d^{-\alpha }[f(x)]}{dx^{-\alpha }}={}_{x_{0}}D_{x}^{-\alpha }f(x)=\frac{1}{\Gamma (\alpha )}\int_{x_{0}}^{x}{\left(x-t\right)}^{\alpha -1}f(t)dt.$$ (1)

The fractional derivative is defined as follows:

$$\frac{d^{\alpha }[f(x)]}{dx^{\alpha }}={}_{x_{0}}D_{x}^{\alpha }f(x)=\frac{d^{m}}{dx^{m}}\left({}_{x_{0}}D_{x}^{-(m-\alpha )}f(x)\right)=\frac{1}{\Gamma (m-\alpha )}\frac{d^{m}}{dx^{m}}\left[\int_{x_{0}}^{x}\frac{f(t)}{{\left(x-t\right)}^{\alpha -m+1}}dt\right].$$ (2)

Where the lower left and lower right indices of ${}_{x_{0}}D{}_{x}$ indicate the ranges of integration, *α* is the order number of the fractional calculus (0 < *α*, *m* − 1 < *α* < *m*, *m* ∈ N*), and Γ is the Gamma function, where $\Gamma (z)=\int_{0}^{\infty }t^{z-1}e^{-t}dt=2\int_{0}^{\infty }t^{2z-1}e^{-t^{2}}dt$, Γ(1 + *z*) = *z*Γ(*z*)(*z* ∈ N*), and Re(*z*) > 0.

The Laplace transform formulas for fractional calculus are as follows:

$$\left\{\begin{array}{l} L\left[{}_{0}D_{t}^{-\alpha }f(t),i\right]=i^{-\alpha }\bar{f}(i)(\alpha >0)\\ L\left[{}_{0}D_{t}^{\alpha }f(t),i\right]=i^{\alpha }\bar{f}(i)(0\leq \alpha \leq 1) \end{array}\right.,$$ (3)

Where the Laplace transform of *f*(*t*) is denoted as $\bar{f}(i)$.

### Fractional element

**Fig 2:**
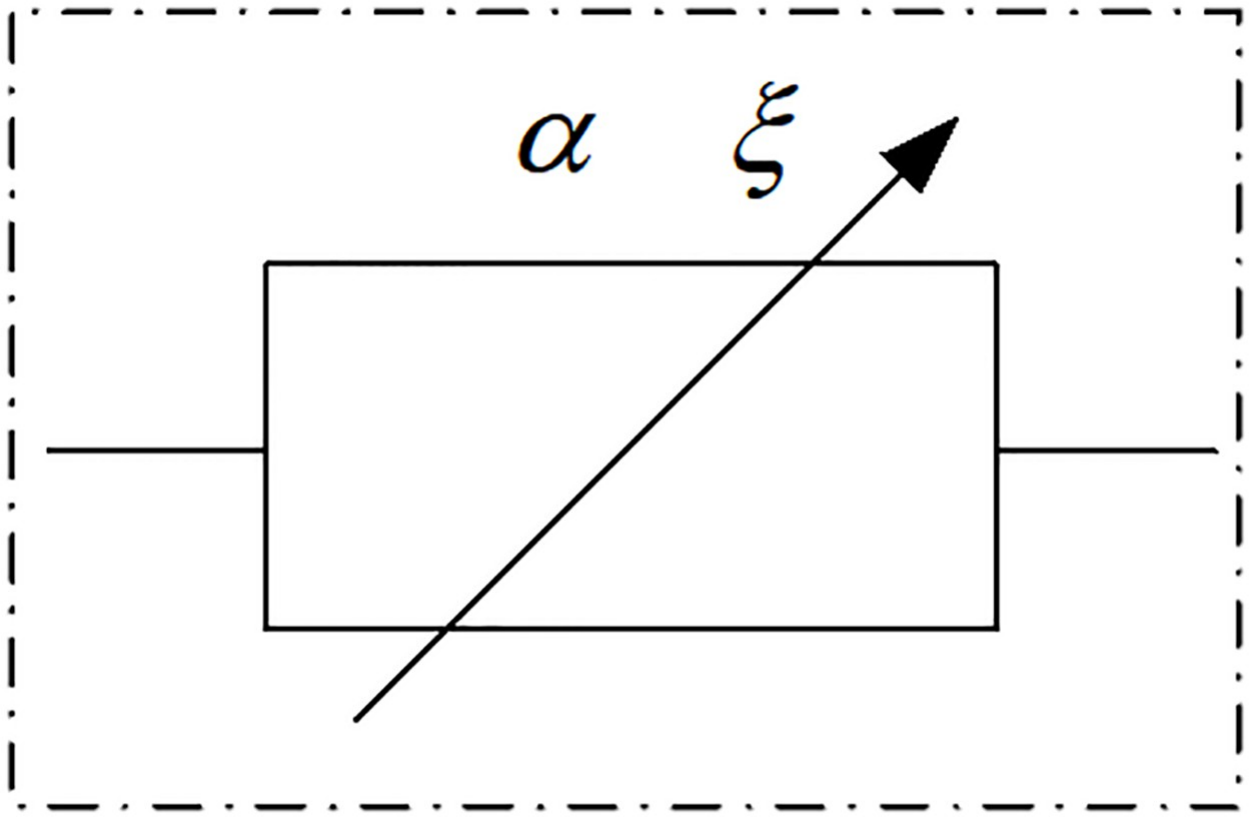
Fractional element.

The state of an object is assumed to be between an ideal solid and an ideal fluid. The relationship between stress and strain is expressed by a fractional derivative, and this object is referred to as a fractional element [32]. The mechanical model is shown in Fig 2.

The constitutive equation of the fractional element is as follows [35, 36]:

$$\sigma (t)=\xi \frac{d^{\alpha }\varepsilon (t)}{dt^{\alpha }},(0\leq \alpha \leq 1).$$ (4)

Where *ξ* is the inherent coefficient of the fractional element. When *α* = 1 and $\sigma (t)=\xi \frac{d\varepsilon (t)}{dt}$, $\frac{d\varepsilon (t)}{dt}$ is the rate of strain, and the fractional element is equivalent to a damper element, which represents an ideal fluid. When *α* = 0 and *σ*(*t*) = *ξε*(*t*), the fractional element is equivalent to a spring element, which represents an ideal solid.

When *σ*(*t*) = *const*, the fractional element describes the creep behavior under the condition of constant stress. Eq (4) is integrated according to Riemann–Liouville fractional calculus theory, and the creep equation of the fractional element can be calculated as follows:

$$\varepsilon (t)=\frac{\sigma }{\xi }\frac{t^{\alpha }}{\Gamma (1+\alpha )},(0<\alpha <1)$$ (5)

**Fig 3:**
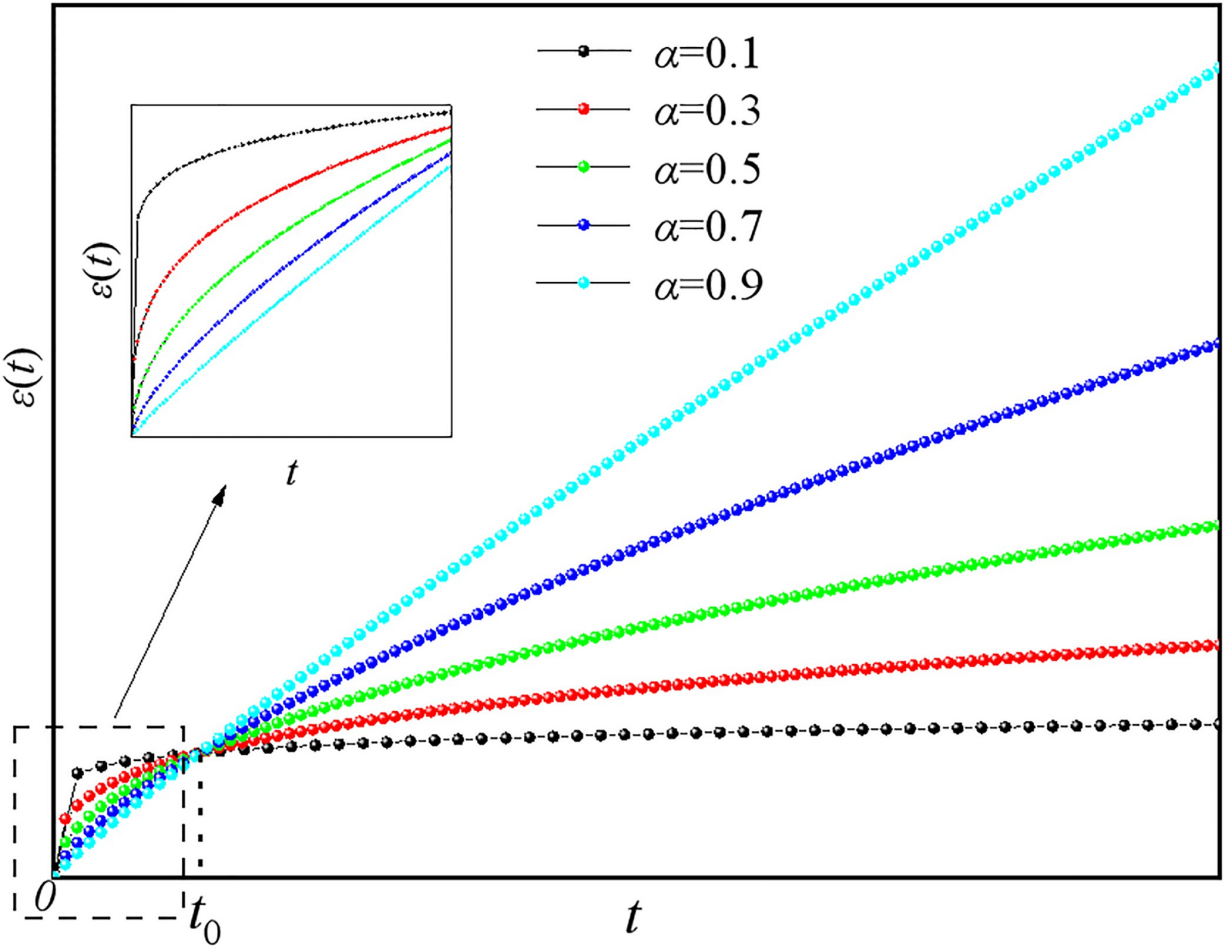
Creep curves of fractional element with different *α* values.

From this, it can be determined that the strain of the fractional element has a power function relationship with time. The creep curves for different *α* values are plotted according to Eq (5) in Fig 3, showing that the fractional element can characterize the initial creep stage.

Similarly, when *ε*(*t*) = *const*, a fractional element will describe the stress relaxation of the creep motion. At this time, the stress of the fractional element changes with time, and the strain remains unchanged. The relaxation equation of the fractional element can be derived as follows:

$$\sigma (t)=\varepsilon \cdot \xi \frac{t^{-\alpha }}{\Gamma (1-\alpha )},0<\alpha <1.$$ (6)

### Viscous element with damage variables

**Fig 4:**
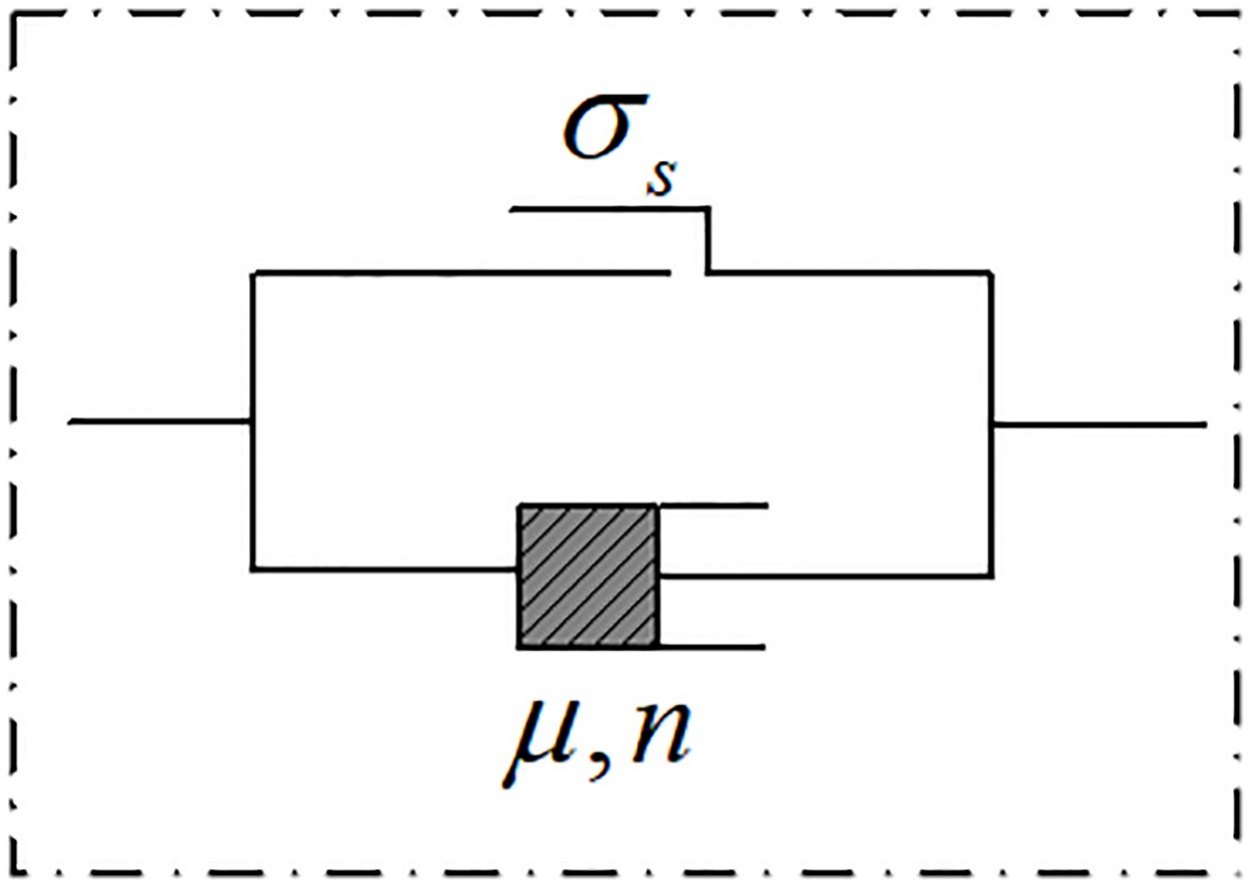
Viscoplastic element with damage variables.

When the backfill materials containing water enter the stage of accelerated creep, internal damage will appear and gradually develop into macroscopic cracks. To accurately describe the creep characteristics of backfill materials in this stage, a viscoplastic element with damage variables is introduced. The mechanical model is shown in Fig 4.

The damage variable first appeared in damage mechanics. The evolution equation of the damage variable proposed by the former Soviet Union scholar Kachanvo is [37]:

$$\dot{D}=C{\left(\frac{\sigma }{1-D}\right)}^{n},$$ (7)

The creep failure time can be obtained by integrating the Eq (7):

$$t_{m}={\left[C\left(1+n\right)\sigma^{n}\right]}^{-1},$$ (8)

Combining Eqs (7) and (8), the evolution equation of damage variable with time is:

$$D=1-{\left(1-\frac{t}{t_{m}}\right)}^{\frac{1}{1+n}},$$ (9)

Therefore, the constitutive equation of the viscous element with damage variables is as follows:

$$\varepsilon_{vp}=\frac{\sigma -\sigma_{s}}{\mu }\frac{t_{m}\left(1+n\right)}{n}\left[1-{\left(1-\frac{t}{t_{m}}\right)}^{\frac{n}{1+n}}\right],$$ (10)

Where *σ*_*s*_ is the yield strength of an object, *σ* is the applied stress, *μ* is the damaged element viscosity coefficient, *n* is the inherent material coefficient, and *t*_*m*_ is the creep failure time of the object.

**Fig 5:**
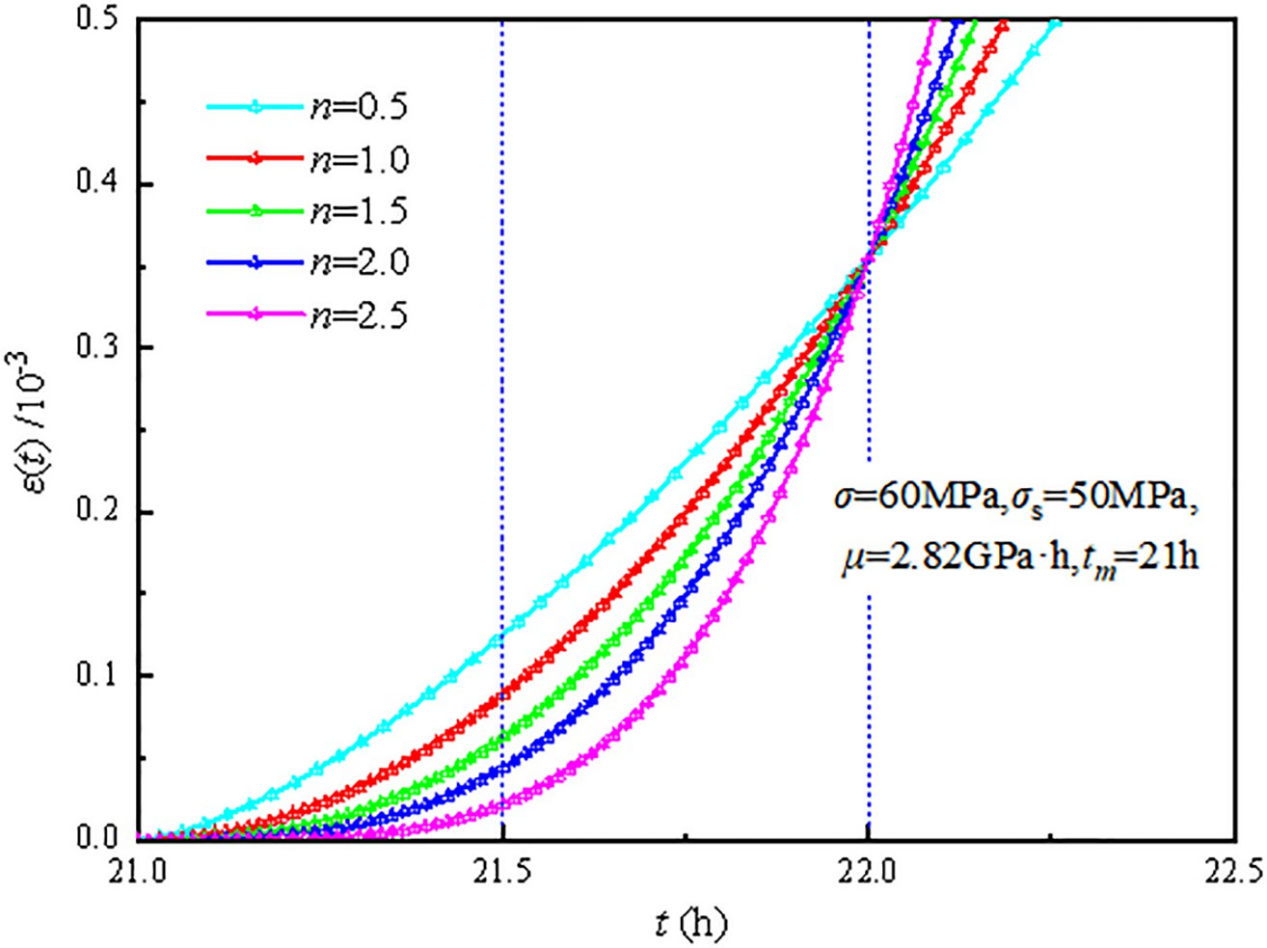
Creep curves of viscous element with damage variables with different *n* value.

The creep curves for different values of *n* when $\left\{\begin{array}{l} \sigma =60\text{MPa},\sigma_{s}=50\text{MPa},\\ \mu =\text{2}.82\text{GPa}\cdot \text{h},t_{m}=21\text{h} \end{array}\right.$ are plotted in Fig 5. As shown in Fig 5, the viscous element with damage variables can theoretically characterize the accelerated creep stage.

## Bingham model

**Fig 6:**
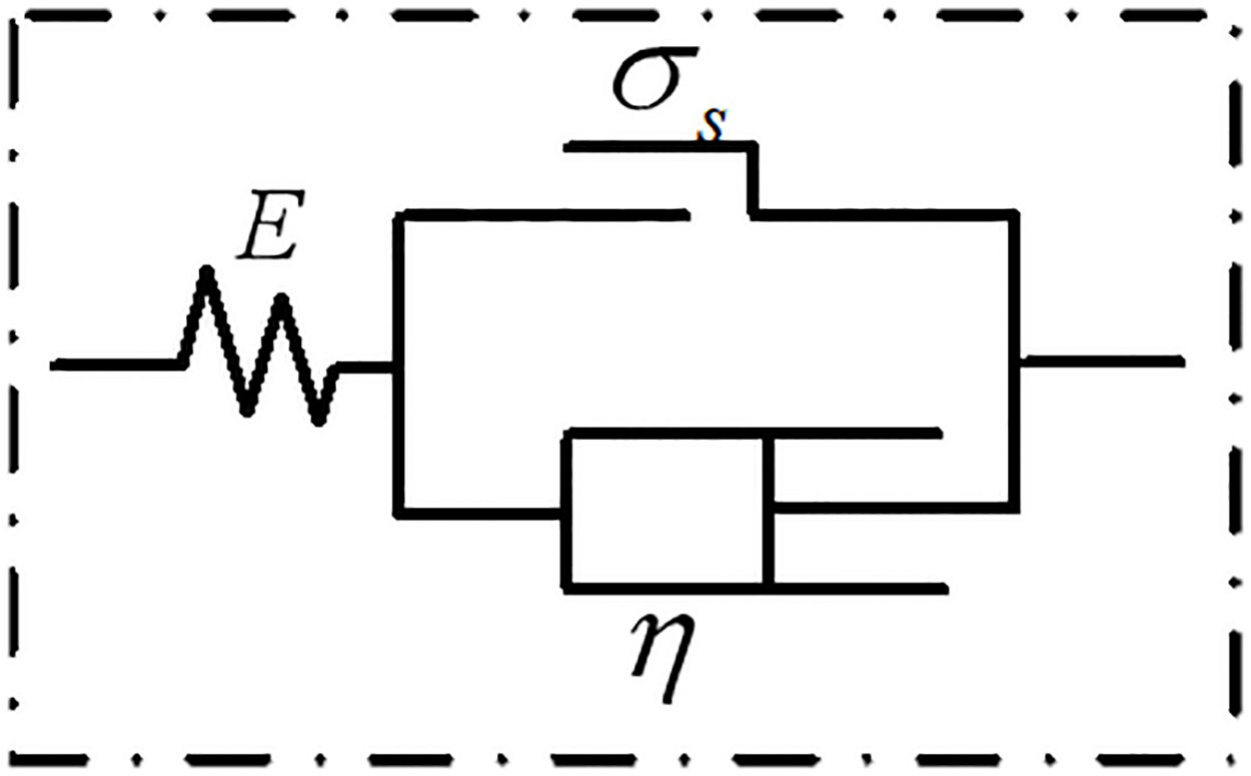
Traditional Bingham model [22].

The traditional Bingham model [22] is composed of a Hooke element and an elastic viscoplastic element in series. The mechanical model is shown in Fig 6.

The constitutive equations are as follows:

$$\sigma <\sigma_{s},\sigma =E\varepsilon ,\dot{\sigma }=E\dot{\varepsilon },$$ (11)

$$\sigma \geq \sigma_{s},\eta \dot{\sigma }+E(\sigma -\sigma_{s})=E\eta \dot{\varepsilon },$$ (12)

Where *ε* is the strain,$\dot{\sigma }$ and $\dot{\varepsilon }$ are the rates of stress and strain, respectively, *E* is the elastic modulus, and *η* is the viscosity coefficient.

## Creep equation of improved Bingham model

### One-dimensional creep equation of improved Bingham model

**Fig 7:**
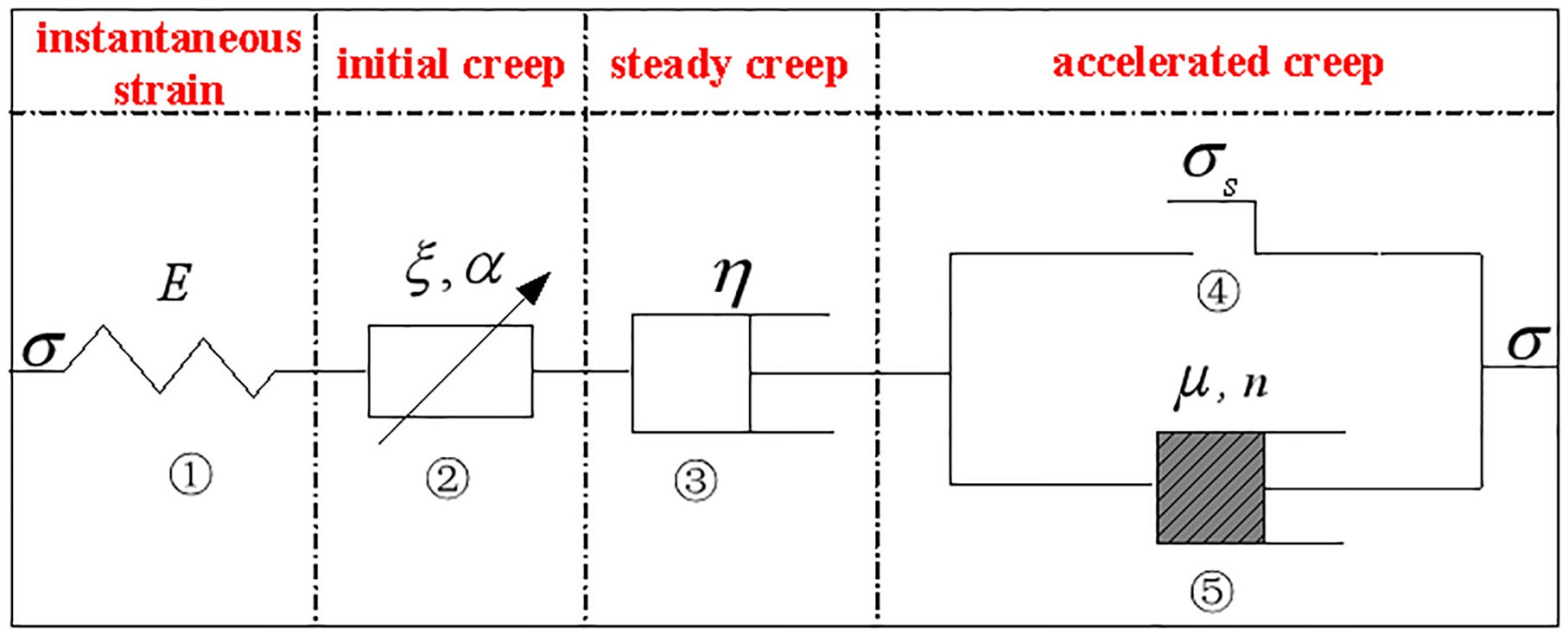
Improved Bingham model.

To overcome the shortcomings of the traditional Bingham model, the fractional element is introduced. The fractional element and viscous element are connected in series with the Bingham model. Then, the viscous element in the Bingham model is replaced by a viscous element with damage variables. An improved five-element Bingham model is proposed. As shown in Fig 7, element ①, element ②, element ③, element ④, and element ⑤ are arranged from left to right.

**Fig 8:**
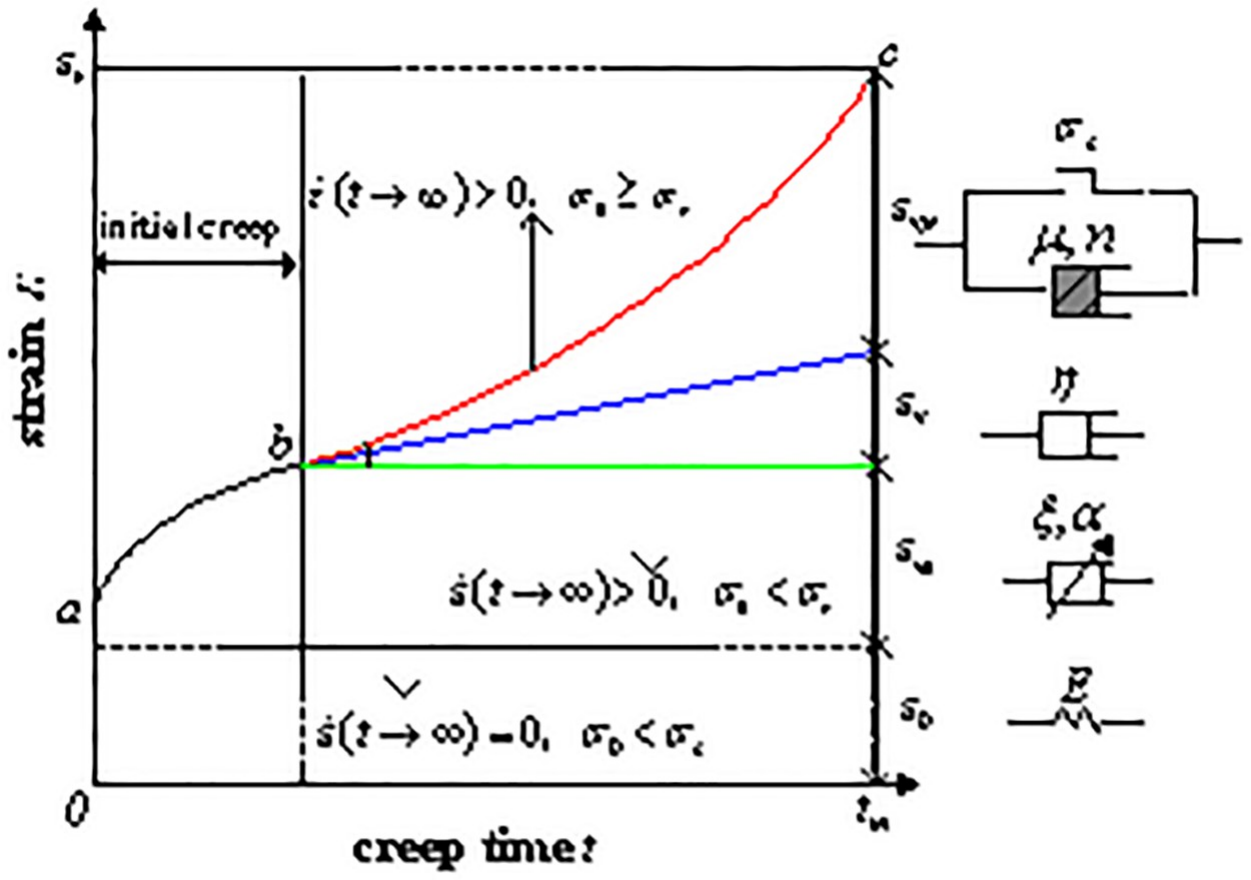
Classical creep curve.

The creep curve is shown in Fig 8. The whole process of creep is divided into three stages: initial creep, steady creep, and accelerated creep. Different elements play their respective roles in different creep stages. The instantaneous strain of the backfill materials mass is described by elastic element ①. Entering the initial creep stage, the relationship between strain and time is nonlinear, and the overall trend is upward convex. The fractional element strain and time followed a power function relationship, and this stage is described by the fractional element ②. If the applied stress does not reach the yield strength of the backfill materials, the stable creep stage is entered. The strain increases linearly with time, and the backfill materials are viscous. This stage is described by the viscous element ③. If the applied stress reaches the yield strength of the backfill materials, the accelerated creep stage is entered. There is a nonlinear relationship between the strain and time. The overall trend is concave downward, and damage occurs inside the backfill materials mass. This stage is described by a viscous element containing damage variables.

The improved creep model satisfies the following conditions:

(1) When $\dot{\varepsilon }\left(t\to \infty \right)=0,\sigma <\sigma_{s}$, the model is equivalent to a Hooke element and a fractional element connected in series. The stress of the backfill materials has not reached its yield strength, and the deformation is in the initial creep stage. According to the principle of elements in series, the stress of each element is the same, and the total strain of the model is equal to the sum of the strain of each element. The following constitutive equation of the improved creep model can be obtained:

$$\varepsilon (t)=\frac{\sigma }{E}+\frac{\sigma }{\xi }\frac{t^{\alpha }}{\Gamma (1+\alpha )},$$ (13)

Where *σ* is the initial stress, and *E* is the elastic modulus.

(2) When $\dot{\varepsilon }\left(t\to \infty \right)>0,\sigma <\sigma_{s}$, the model is equivalent to a Hooke element, fractional element, and viscous element connected in series. The creep reaches the stage of steady creep. The constitutive equation of the creep model is

$$\varepsilon (t)=\frac{\sigma }{E}+\frac{\sigma }{\xi }\frac{t^{\alpha }}{\Gamma (1+\alpha )}+\frac{\sigma }{\eta }t.$$ (14)

(3) When $\dot{\varepsilon }\left(t\to \infty \right)>0,\sigma \geq \sigma_{s}$, all parts of the model are involved in the creep. According to the superposition principle, the constitutive equation of the improved creep model is as follows:

$$\varepsilon (t)=\frac{\sigma }{E}+\frac{\sigma }{\xi }\frac{t^{\alpha }}{\Gamma (1+\alpha )}+\frac{\sigma }{\eta }t+\frac{\sigma -\sigma_{s}}{\mu }\frac{t_{m}\left(1+n\right)}{n}\left[1-{\left(1-\frac{t}{t_{m}}\right)}^{\frac{n}{1+n}}\right].$$ (15)

By combining Eqs (13)–(15), the fractional viscoelastic–plastic one-dimensional creep model expression is obtained as follows:

$$\varepsilon (t)=\left\{\begin{array}{l} \frac{\sigma }{E}+\frac{\sigma }{\xi }\frac{t^{\alpha }}{\Gamma (1+\alpha )},\left(\dot{\varepsilon }(t\to \infty )=0,\sigma <\sigma_{s}\right)\\ \frac{\sigma }{E}+\frac{\sigma }{\xi }\frac{t^{\alpha }}{\Gamma (1+\alpha )}+\frac{\sigma }{\eta }t,\left(\dot{\varepsilon }(t\to \infty )>0,\sigma <\sigma_{s}\right)\\ \frac{\sigma }{E}+\frac{\sigma }{\xi }\frac{t^{\alpha }}{\Gamma (1+\alpha )}+\frac{\sigma }{\eta }t+\frac{\sigma -\sigma_{s}}{\mu }\frac{t_{m}\left(1+n\right)}{n}\left[1-{\left(1-\frac{t}{t_{m}}\right)}^{\frac{n}{1+n}}\right],\left(\dot{\varepsilon }(t\to \infty )>0,\sigma \geq \sigma_{s}\right) \end{array}\right..$$ (16)

### Three-dimensional creep equation of improved Bingham model

In actual working conditions, such as backfill materials mining and excavation, backfill materials are mainly in a three-dimensional stress state. To allow the model to have a certain practical reference significance in practical engineering, the creep equation under the three-dimensional stress state is now deduced. The theoretical basis for previous work is deduced [38], and the following assumptions are made:

1. backfill materials is an isotropic material with consistent damage in all directions.
2. Damage occurs only in the accelerated creep stage and does not occur in other stages of creep.
3. The damage duration is consistent with the corresponding creep time.

Under the three-dimensional stress state, it is assumed that the total strain produced by the creep of backfill materials is $\varepsilon_{ij}^{t}$. The strain of the elastic element is $\varepsilon_{ij}^{e}$, the strain of the fractional element is $\varepsilon_{ij}^{f}$, the strain of the viscous element is $\varepsilon_{ij}^{v}$, and the strain of the viscous element with a damage variable is $\varepsilon_{ij}^{vp}$. According to the superposition principle,

$$\varepsilon_{ij}^{t}=\varepsilon_{ij}^{e}+\varepsilon_{ij}^{f}+\varepsilon_{ij}^{v}+\varepsilon_{ij}^{vp}.$$ (17)

According to elastic mechanics, the elastic element has the following relationship. The stress tensor *σ*_*ij*_ can be divided into a spherical stress tensor *σ*_*m*_*δ*_*ij*_ and a deviatoric stress tensor *S*_*ij*_. *σ*_*m*_ is the average stress in three directions. The strain tensor *ε*_*ij*_ can be divided into a spherical strain tensor *ε*_*m*_*δ*_*ij*_ and a deviatoric strain tensor *e*_*ij*_. *ε*_*m*_ is the average strain in three directions. Thus, the following relationship holds:

$$\left\{\begin{array}{l} \sigma_{ij}=S_{ij}+\delta_{ij}\sigma_{m}\\ \varepsilon_{ij}=e_{ij}+\delta_{ij}\varepsilon_{m} \end{array}\right.,$$ (18)

where *δ*_*ij*_ is the Kronecker tensor [39], which is $\delta_{ij}=\left\{\begin{array}{l} 1,i=j\\ 0,i\neq j \end{array}\right.$.

According to the generalized Hooke’s law,

$$\left\{\begin{array}{l} \sigma_{m}=3K_{1}\varepsilon_{m}\\ S_{ij}=2G_{1}e_{ij} \end{array}\right.,$$ (19)

where *K*_1_ is the bulk modulus, and *G*_1_ is the shear modulus.

Therefore, the creep equation of an elastic element under a three-dimensional stress state is

$$\varepsilon_{ij}^{e}=\frac{1}{2G_{1}}S_{ij}+\frac{1}{3K_{1}}\delta_{ij}\sigma_{m}.$$ (20)

The spherical stress tensor has little effect on creep, ignoring the effect of spherical stress tensor on creep. The creep equation of a fractional element under a three-dimensional stress state is

$$\varepsilon_{ij}^{f}=\frac{S_{ij}}{\xi }\cdot \frac{t^{\alpha }}{\Gamma (1+\alpha )}.$$ (21)

The creep equation of a viscous element under a three-dimensional stress state is

$$\varepsilon_{ij}^{v}=\frac{S_{ij}}{2\eta }t.$$ (22)

The creep equation of the viscous element with damage variables under the three-dimensional stress state is

$$\varepsilon_{vp}=\frac{t_{m}\left(1+n\right)}{2\mu n}\cdot \left[1-{\left(1-\frac{t}{t_{m}}\right)}^{\frac{n}{1+n}}\right]\langle \Phi \left(\frac{F}{F_{0}}\right)\rangle \frac{\partial Q}{\partial \sigma_{ij}},$$ (23)

Where $\langle \Phi \left(\frac{F}{F_{0}}\right)\rangle =\left\{\begin{array}{l} \Phi \left(\frac{F}{F_{0}}\right),\left(F\geq 0\right)\\ 0,\left(F<0\right) \end{array}\right.$, *F* is the backfill materials yield function, *F*_0_ is the initial value of the backfill materials yield function, which is usually set to 1, $\Phi \left(\frac{F}{F_{0}}\right)={\left(\frac{F}{F_{0}}\right)}^{\psi }$, *ψ* is a constant, which is usually set to 1, and *Q* is the plastic potential function. According to the associated flow law, *Q* = *F*.

According to the superposition principle, the three-dimensional creep equation is obtained as follows:

$$\varepsilon_{ij}^{t}=\left\{\begin{array}{l} \frac{S_{ij}}{2G_{1}}+\frac{\delta_{ij}\sigma_{m}}{3K_{1}}+\frac{S_{ij}}{\xi }\frac{t^{\alpha }}{\Gamma (1+\alpha )}+\frac{S_{ij}}{2\eta }t\;F<0\\ \frac{S_{ij}}{2G_{1}}+\frac{\delta_{ij}\sigma_{m}}{3K_{1}}+\frac{S_{ij}}{\xi }\frac{t^{\alpha }}{\Gamma (1+\alpha )}+\frac{S_{ij}}{2\eta }t+\frac{t_{m}\left(1+n\right)F}{2\mu n}\left[1-{\left(1-\frac{t}{t_{m}}\right)}^{\frac{n}{1+n}}\right]\frac{\partial F}{\partial \sigma_{ij}}\;F\geq 0 \end{array}\right.$$ (24)

Based on the viscoelastic–plastic model, the Drucker–Prager yield criterion [40] is adopted, and its equation is

$$F=\sqrt{J_{2}}-bI_{1}-a,$$ (25)

Where *J*_2_ is the stress deviator second invariant, *I*_1_ is the first invariant of the stress tensor, and *a* and *b* are material parameters. *a* and *b* are defined as follows:

$$\left\{\begin{array}{l} a=\frac{\text{sin}\;\theta }{\sqrt{3}\sqrt{3+{\text{sin}}^{2}\;\theta }}\\ b=\frac{\sqrt{3}c\cdot \text{cos}\;\theta }{\sqrt{3+{\text{sin}}^{2}\;\theta }} \end{array}\right.,$$ (26)

Where *θ* is the internal friction angle of the backfill materials, and *c* is the cohesive force of the backfill materials.

In the three-dimensional axially creep test of backfill materials, the following relationships are satisfied:

$$\left\{\begin{array}{l} \sigma_{1}>\sigma_{2}=\sigma_{3};\\ \sigma_{m}=\frac{\sigma_{1}+\sigma_{2}+\sigma_{3}}{3};\\ \sqrt{J_{1}}=\frac{\sigma_{1}-\sigma_{3}}{\sqrt{3}};\\ I_{1}=\sigma_{1}+\sigma_{2}+\sigma_{3};\\ S_{11}=\sigma_{1}-\sigma_{m};\\ \frac{\partial F}{\partial \sigma_{11}}=\frac{\sqrt{3}-3b}{3} \end{array}\right.$$ (27)

By combining Eqs (17)–(27), according to the superposition principle, the viscoelastic–plastic damage axial creep equation of the backfill materials containing water under the three-dimensional stress state is obtained as follows:

$$\varepsilon_{11}^{t}=\left\{\begin{array}{l} \frac{\sigma_{1}-\sigma_{3}}{3G_{1}}+\frac{\sigma_{1}+2\sigma_{3}}{9K_{1}}+\frac{2(\sigma_{1}-\sigma_{3})}{3\xi }\frac{t^{\alpha }}{\Gamma (1+\alpha )},\left(\dot{\varepsilon }\left(t\to \infty \right)=0,\sigma_{0}<\sigma_{s}\right);\\ \frac{\sigma_{1}-\sigma_{3}}{3G_{1}}+\frac{\sigma_{1}+2\sigma_{3}}{9K_{1}}+\frac{2(\sigma_{1}-\sigma_{3})}{3\xi }\frac{t^{\alpha }}{\Gamma (1+\alpha )}+\frac{\sigma_{1}-\sigma_{3}}{3\eta }t,\left(\dot{\varepsilon }\left(t\to \infty \right)>0,\sigma_{0}<\sigma_{s}\right);\\ \frac{\sigma_{1}-\sigma_{3}}{3G_{1}}+\frac{\sigma_{1}+2\sigma_{3}}{9K_{1}}+\frac{2(\sigma_{1}-\sigma_{3})}{3\xi }\frac{t^{\alpha }}{\Gamma (1+\alpha )}+\frac{\sigma_{1}-\sigma_{3}}{3\eta }t+\left(\frac{\sqrt{3}-3b}{3}\right)\frac{t_{m}\left(1+n\right)}{2\mu n}\times \cdots \\ \left[1-{\left(1-\frac{t}{t_{m}}\right)}^{\frac{n}{1+n}}\right]\left(\sigma_{1}-\sigma_{3}-\sigma_{s}\right),\left(\dot{\varepsilon }\left(t\to \infty \right)>0,\sigma_{0}\geq \sigma_{s}\right); \end{array}\right.$$ (28)

## Parameter identification and model validation

### Parameter identification

(1) Determination of *G*_1_ and *K*_1_

In Eq (28),

$$\left\{\begin{array}{l} G_{1}=\frac{E}{2\left(1+\nu \right)};\\ K_{1}=\frac{E}{3\left(1-2\nu \right)} \end{array}\right.$$ (29)

The elastic modulus *E* and Poisson’s ratio *ν* were measured by uniaxial compression tests, and *G*_1_ and *K*_1_ were obtained by substituting into the above equation.

(2) Determination of *ξ* and *α*

The data in the initial creep stage were fitted with the following nonlinear functions:

$$f\left(t_{i}\right)=a_{0}t_{i}^{b_{0}}+c_{0},$$ (30)

$$\left\{\begin{array}{l} f\left(t_{i}\right)=\varepsilon \left(t_{i}\right);\\ a_{0}=\frac{2(\sigma_{1}-\sigma_{3})}{3\xi \cdot \Gamma \left(1+\alpha \right)};\\ b_{0}=\alpha ;\\ c_{0}=\frac{\sigma_{1}-\sigma_{3}}{3G_{1}}+\frac{\sigma_{1}+2\sigma_{3}}{9K_{1}} \end{array}\right.$$ (31)

The parameters of the model were obtained simultaneously as follows:

$$\left\{\begin{array}{l} \alpha =b_{0};\\ \xi =\frac{2(\sigma_{1}-\sigma_{3})}{3a_{0}\cdot \Gamma \left(1+b_{0}\right)} \end{array}\right.$$ (32)

The Γ function value was calculated by the Maple software [41].

(3) Determination of *η*

The data in the steady creep stage were fitted with linear functions:

$$f\left(t_{i}\right)=a_{1}t_{i}+c_{1},$$ (33)

$$\left\{\begin{array}{l} f\left(t_{i}\right)=\varepsilon \left(t_{i}\right);\\ a_{1}=\frac{\sigma_{1}-\sigma_{3}}{3\eta }; \end{array}\right.$$ (34)

The parameters of the model were obtained simultaneously, and the following formula was applied:

$$\eta =\frac{\sigma_{1}-\sigma_{3}}{3a_{1}}.$$ (35)

(4) Determination of *t*_*m*_, *μ*, and *n*

*t*_*m*_ was obtained from the creep test, and the parameters *μ* and *n* are obtained by the following method. It was assumed that the functional relationship between the strain and parameters was *ε*_*i*_ = *f*(*t*_*i*_, *μ*, *n*). There were a total of *m* groups of experimental data (*t*_*k*_, *ε*_*k*_)(*k* = 1,2,3,⋯,*m*). First, a set of initial values of parameters *μ*_0_ and *n*_0_ was specified, so that the initial parameters of the model were determined. Each value of *t*_*k*_ was substituted into the creep equation to obtain the theoretical value ${\tilde{\varepsilon }}_{k}$. To minimize the error, the calculation method reported previously was adopted [42]. When $\sum_{k=1}^{m}{\left[\varepsilon_{k}-{\tilde{\varepsilon }}_{k}\right]}^{2}$ was the smallest, the following conditions must be met:

$$\left\{\begin{array}{l} \frac{\partial \sum_{k=1}^{m}{\left[\varepsilon_{k}-{\tilde{\varepsilon }}_{k}\right]}^{2}}{\partial \mu_{0}}=0;\\ \frac{\partial \sum_{k=1}^{m}{\left[\varepsilon_{k}-{\tilde{\varepsilon }}_{k}\right]}^{2}}{\partial n_{0}}=0. \end{array}\right.$$ (36)

When using this method, iterative divergence will occur if the initial value is not selected properly. Therefore, a "damping factor" *d* was added to ensure the method iteratively converged [38].

### Model validation

To deeply explore the effect of water on the mechanical properties of backfill materials, triaxial creep experiments of backfill materials under different moisture contents were conducted. The experimental data came from the triaxial creep test of gangue cemented filling material under different moisture contents. The detailed experimental procedure was reported previously [33].

According to the test procedure of the International Society for Rock Mechanics (ISRM), a standard cylinder sample with a diameter of 50 mm and a height-to-diameter ratio of 2:1 was prepared. To analyze the influence of the moisture content changes on the mechanical properties of the backfill materials, four groups of samples with moisture contents of 0% (dry), 8.8%, 17.6%, and 22% (completely saturated) were prepared by using a vacuum saturator and a drying oven, respectively. There were 24 samples in each group of six. The long-term strengths of the samples with 0%, 8.8%, 17.6%, and 22% moisture contents were 7.19, 6.22, 4.62 and 4.14 MPa, respectively. The composition of gangue cemented filling material is shown in Table 1. The basic physical and mechanical parameters of the samples under different moisture contents are shown in Table 2.

**Table 1:** Main elements of cemented fillers [33].

Main elements	Coal gangue (%)	Coal fly ash (%)	Cement (%)
**Quartz**	51.7	9.9	/
**Dahllite**	/	/	67.6
**Feldspar**	13.6	/	/
**Kaolinite**	25.1	/	/
**Greenite**	/	20.1	/
**Alumina**	/	48.2	/
**Kyanite**	/	/	11.6
**Other**	9.6	10.7	8.1

**Table 2:** Basic physical and mechanical parameters of samples with different moisture contents [33].

Moisture content (%)	Poisson’s ratio	Uniaxial compressive strength (MPa)	Elastic modulus (GPa)	Cohesion (MPa)	Internal friction angle (°)
0.0	0.21	10.41	0.355	2.63	22
8.8	0.24	9.81	0.261	2.49	25
17.6	0.28	8.82	0.213	2.36	27
22.0	0.34	8.31	0.182	2.13	30

The MTS 815.02 electro-hydraulic servo-controlled rock mechanics testing system was used in the test equipment, and the maximum axial compression can reach 1700 kN. The compressive axial load rate can be controlled within 10^−5^~1 mm/s. The equipment can carry out creep tests.

**Fig 9:**
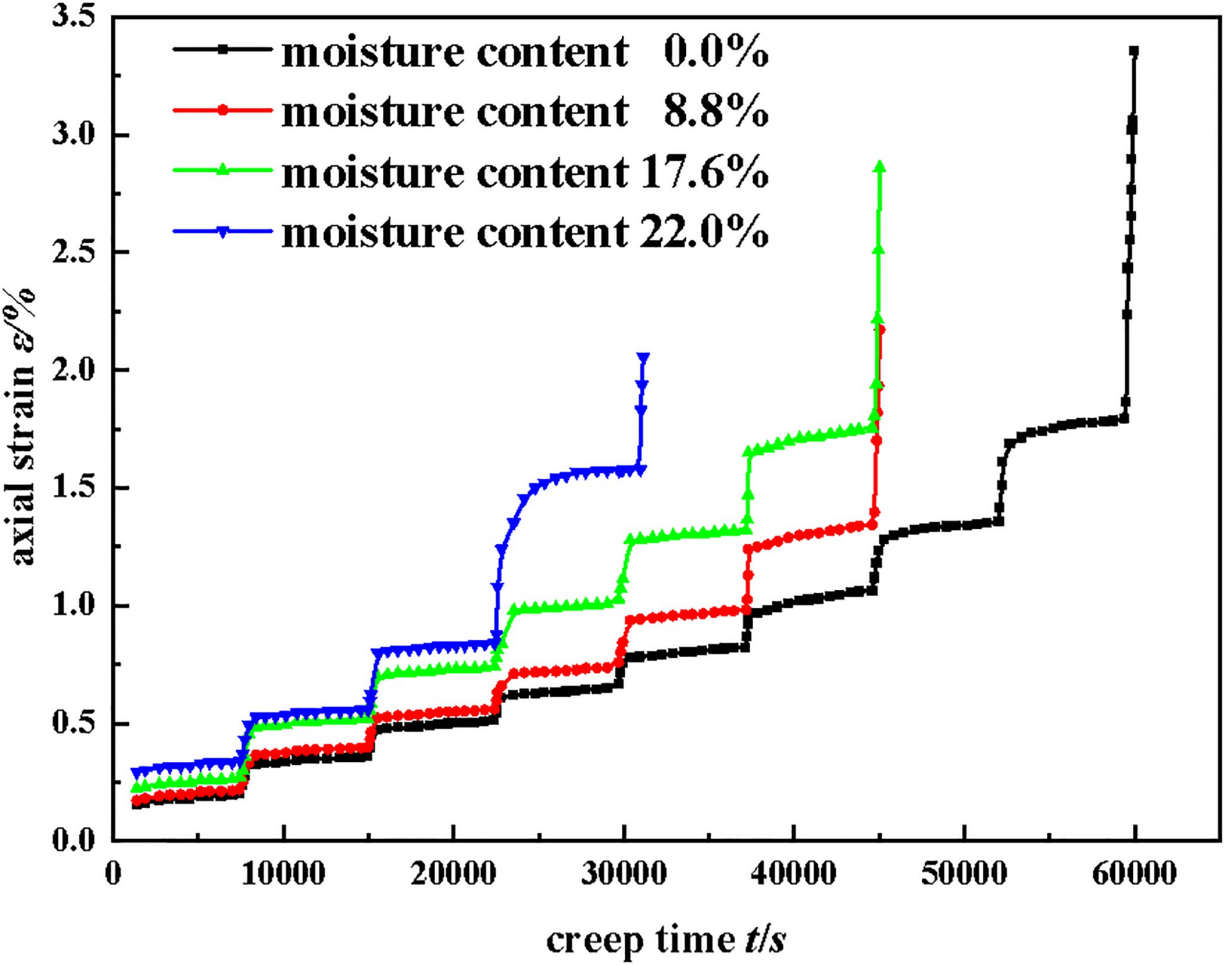
Creep curves of backfill materials with different moisture contents [33].

In this test, the confining pressure was first loaded to 1 MPa, and then the confining pressure was fixed. Axially controlled loading was then applied. The loading rate was 0.02 kN/s, the incremental load was 4 kN (2.04 MPa) for each stage, and the next stage was loaded every 7200 s until the specimen failed. After the specimen was destroyed, the confining pressure was first unloaded to zero, then the axial load was unloaded to zero, and finally, the test data was derived. Fig 9 shows a graph of the results of the triaxial creep test performed on the sample. Accelerated creep occurred when the moisture content of the sample was 0% and the axial stress was 18.36 MPa, and the moisture content was 8.8% and the axial stress was 14.28 MPa. The moisture content of 17.8% and 22% did not show the accelerated creep stage, because the interval of axial stress was large, and the failure occurred before reaching the next axial stress.

As can be seen in Fig 9, with the increase of axial stress, the total deformation of the specimen increased gradually under the condition of a certain moisture content. Under the action of low axial stress, the specimen only had immediate deformation and initial creep stage. With the increase of axial stress, the specimen gradually underwent a constant creep stage and accelerated creep stage. It showed that the creep rate increased with the increase of axial stress. Under certain axial stress, with the increase of moisture content, the total deformation of the specimen increased gradually, and the total creep time decreased gradually. It showed that the moisture content had an obvious deterioration effect on the specimen, which greatly accelerated the creep failure process of the specimen.

**Fig 10:**
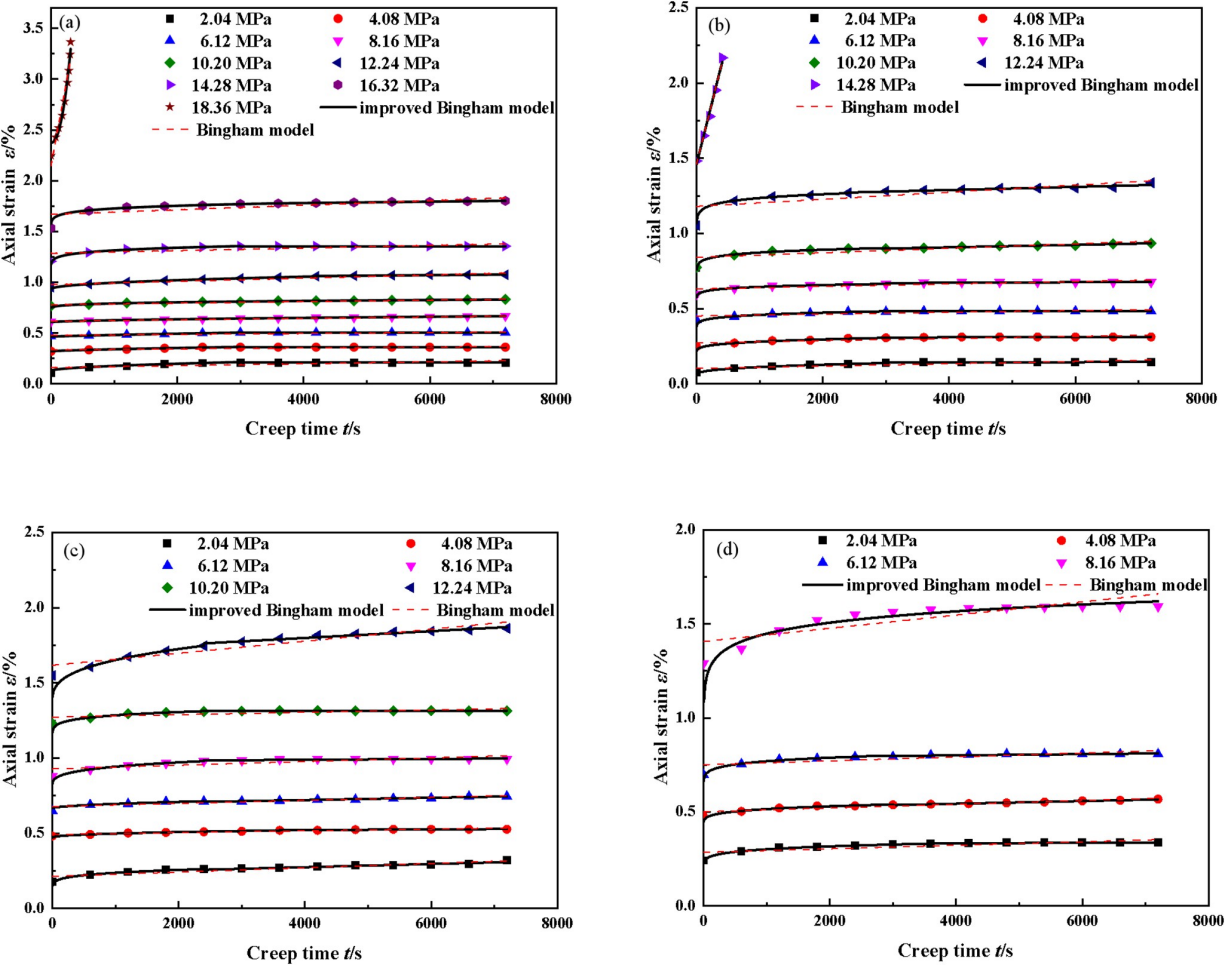
Fitted creep curves of backfill materials with different moisture contents [33]. (a)0.00%(b)8.8%(c)17.6%(d)22.0%.

According to the experimental results, using the parameter inversion method, the inversion results of the creep parameters are shown in Table 3. When the confining pressure was 1 MPa and the moisture content was 0%, the constant-velocity creep rate of backfill materials was basically 0 at the first six stress levels. At this time, the first formula of Eq (28) was used. Under the 7th and 8th stress levels, the backfill materials strain included not only elastic and viscoelastic strains but also viscous strains. The second formula of Eq (28) was used. The long-term strength of backfill materials exceeded the 9th stress level. The third formula of Eq (28) was used. The results shown in Fig 10(a)–10(d) show the comparisons of the experimental and theoretical curves when the moisture content was 0.0%, 8.8%, 17.6%, and 22%, respectively. The degree of agreement between the experimental curve and the theoretical curve was very high, both above 0.9. Because the test curve of the sample was smoother and fluctuated less under static load conditions, the theoretical curve of the model was basically consistent with the test curve. At the same time, the improved Bingham fractional creep model could accurately describe the initial and constant-velocity creep characteristics of backfill materials containing water at low-stress levels. It can also describe the accelerated creep characteristics of backfill materials containing water under high-stress states. Compared with the traditional Bingham model, the improved model involved a more comprehensive analysis and allowed for more convenient parameter inversion. The creep characteristics of different stages of the deformation of backfill materials containing water can be described by the creep model established in this paper, and the applicability of the model was verified. The traditional Bingham model can only describe the steady creep stage, which indicates that the improved Bingham model in this paper was successful.

**Table 3:** Inversion results of creep parameters.

**Axial stress(MPa)**	**Model parameters of moisture content 0.0%**							
	*G*_1_ (GPa)	*K*_1_ (GPa)	*ξ* (GPa·h^*α*^)	*α*	*η* (10^6^ MPa·h)	*t*_*m*_(h)	*μ* (MPa·h)	*n*
2.04	0.142	0.237	0.36	0.012	0	/	/	/
4.08			1.62	0.016	0	/	/	/
6.12			2.73	0.028	0	/	/	/
8.16			4.84	0.029	0	/	/	/
10.20			6.95	0.042	0	/	/	/
12.24			9.64	0.066	0	/	/	/
14.28			11.32	0.084	3.94	/	/	/
16.32			13.28	0.091	7.73	/	/	/
18.36			14.12	0.126	14.86	16.55	0.256	1.198
**Axial stress(MPa)**	**Model parameters of moisture content 8.8%**							
	*G*_1_ (GPa)	*K*_1_ (GPa)	*ξ* (GPa·h^*α*^)	*α*	*η* (10^6^ MPa·h)	*t*_*m*_(h)	*μ* (MPa·h)	*n*
2.04	0.104	0.174	0.94	0.034	0	/	/	/
4.08			3.67	0.056	0	/	/	/
6.12			8.52	0.085	0	/	/	/
8.16			11.36	0.091	0	/	/	/
10.20			15.58	0.134	6.76	/	/	/
12.24			19.22	0.155	15.34	/	/	/
14.28			25.34	0.183	25.89	12.46	0.354	0.512
**Axial stress(MPa)**	**Model parameters of moisture content 17.6%**							
	*G*_1_ (GPa)	*K*_1_ (GPa)	*ξ* (GPa·h^*α*^)	*α*	*η* (10^6^ MPa·h)	*t*_*m*_(h)	*μ* (MPa·h)	*n*
2.04	0.085	0.142	1.27	0.052	0	/	/	/
4.08			4.63	0.125	0	/	/	/
6.12			9.66	0.134	0	/	/	/
8.16			18.56	0.167	8.84	/	/	/
10.20			24.65	0.224	18.62	/	/	/
12.24			37.64	0.326	37.57	12.41	/	/
**Axial stress(MPa)**	**Model parameters of moisture content 22.0%**							
	*G*_1_ (GPa)	*K*_1_ (GPa)	*ξ* (GPa·h^*α*^)	*α*	*η* (10^6^ MPa·h)	*t*_*m*_(h)	*μ* (MPa·h)	*n*
2.04	0.073	0.121	3.64	0.135	0	/	/	/
4.08			15.44	0.286	10.22	/	/	/
6.12			41.68	0.314	27.39	/	/	/
8.16			60.10	0.410	46.44	8.65	/	/

### Relationship between parameters of model and moisture content

**Fig 11:**
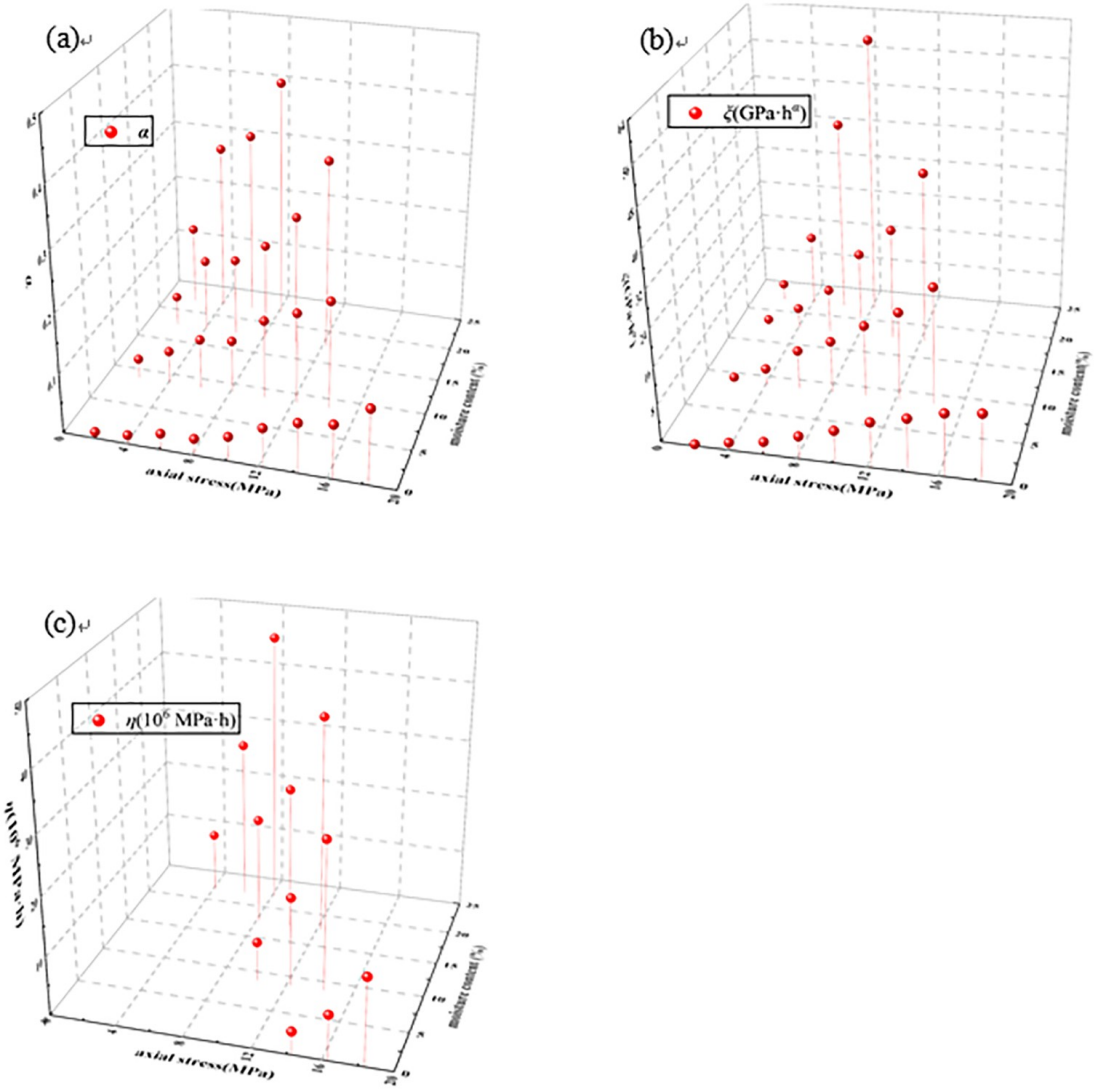
Fitted curves of parameters *ξ*, *α*, *η* and moisture content. (a) *ξ* (b) *α* (c) *η*.

According to the data of Table 2, the relationship between parameters *ξ*, *α*, *η* and moisture content and axial stress is drawn, as shown in Fig 11.

It can be seen from Fig 11 that under the same axial stress, *α* increases gradually with the increase of moisture content. Under the same moisture content, *α* gradually increases with the increase of axial stress. In general, *α* increases with the increase of axial stress and moisture content. Under the same axial stress, *ξ* increases gradually with the increase of moisture content. Under the same moisture content, *ξ* gradually increases with the increase of axial stress. In general, *ξ* increases with the increase of axial stress and moisture content. Under the same moisture content, *η* gradually increases with the increase of axial stress.

## Conclusion

In the study, based on Riemann-Liouville fractional calculus and damage mechanics theory, fractional element and viscous element with damage variables are introduced, so as to solve the problem that the traditional Bingham model is unable to characterize the nonlinear creep process, and then to achieve the purpose of describing the whole process of backfill material creep. The conclusions are drawn below:

1. Fractional element based on the Riemann-Liouville fractional calculus are proposed to provide a better description of the initial creep stage. Viscous element with damage variables based on the theory of damage mechanics can provide a better description of the accelerated creep stage.
2. Based on the superposition principle and Drucker–Prager yield criterion, the one-dimensional and three-dimensional creep equations of the model are derived, and the model parameter inversion method is given. The model parameter *α* and *ξ* increase with the increase of axial stress and moisture content. Under the same moisture content, *η* gradually increases with the increase of axial stress.
3. The improved Bingham fractional creep model can describe the whole creep process of gangue cemented filling material under different moisture contents, especially in the accelerated creep stage, which has a high degree of agreement.
4. The moisture content is not introduced into the creep equation, which is also the next research direction. Moreover, the triaxial creep tests with different confining pressures will be considered to reveal the influence of confining pressure on model parameters in future experimental studies. It is significant to derive the three-dimensional difference form of the fractional creep damage model, so as to be applied to the secondary development of the constitutive relation module of the numerical analysis software.

## Supporting information

S1 Data (ZIP)

## Abbreviations

$\dot{\varepsilon }$: the rates of One-dimensional axial strain
$\dot{\sigma }$: the rates of One-dimensional axial stress
${}_{x_{0}}D_{x}$: the Riemann-Liouville fractional-order integral operator
$\varepsilon_{ij}^{e}$: the strain of the elastic element
$\varepsilon_{ij}^{f}$: the strain of the fractional element
$\varepsilon_{ij}^{vp}$: the strain of the viscous element with a damage variable
$\varepsilon_{ij}^{v}$: the strain of the viscous element
$\varepsilon_{ij}^{t}$: the total strain
*a* and *b*: the material parameters
*c*: the cohesive force of the backfill materials
*E*: the elastic modulus
*e*_*ij*_: the deviatoric strain tensor
*F*: the backfill materials yield function
*G*_1_: the shear modulus
*I*_1_: the first invariant of the stress tensor
*J*_2_: the stress deviator second invariant
*K*_1_: the bulk modulus
*n*: the inherent material coefficient
*Q*: the plastic potential function
*S*_*ij*_: the deviatoric stress tensor
*t*_*m*_: the creep failure time of the object
*α*: the order number of the fractional calculus
Γ: the Gamma function
*δ*_*ij*_: the Kronecker tensor
*ε*: the One-dimensional axial strain
*ε*_*ij*_: the strain tensor
*ε*_*m*_: the average strain in three directions
*η*: the viscosity coefficient
*θ*: the internal friction angle of the backfill materials
*μ*: the damaged element viscosity coefficient
*ξ*: the the inherent coefficient of the fractional element
*σ*: the One-dimensional axial stress
*σ*_*ij*_: the stress tensor
*σ*_*m*_: the average stress in three directions
*σ*_*s*_: the yield strength of an object
*ψ*: the constant
